# Decellularized In Vitro Capillaries for Studies of Metastatic Tendency and Selection of Treatment

**DOI:** 10.3390/biomedicines10020271

**Published:** 2022-01-26

**Authors:** Outi Huttala, Desiree Loreth, Synnöve Staff, Minna Tanner, Harriet Wikman, Timo Ylikomi

**Affiliations:** 1 Cell Biology, Faculty of Medicine and Health Technology, Tampere University, 33100 Tampere, Finland; timo.ylikomi@tuni.fi; 2 Tays Cancer Center, Tampere University Hospital, 33520 Tampere, Finland; synnove.staff@pshp.fi (S.S.); minna.tanner@pshp.fi (M.T.); 3 Department of Tumor Biology, University Medical Center Hamburg-Eppendorf, 20246 Hamburg, Germany; d.loreth@uke.de (D.L.); h.wikman@uke.de (H.W.); 4 Department of Obstetrics and Gynecology, Tampere University Hospital, 33520 Tampere, Finland; 5 Department of Oncology, Tampere University Hospital, 33520 Tampere, Finland; 6 Department of Oncology, Faculty of Medicine and Health Technology, Tampere University, 33100 Tampere, Finland

**Keywords:** tumor microenvironment, cancer, metastatic niches, vasculature, patient-derived cancer cells drug screening, human adipose stromal cells, human umbilical vein endothelial cells

## Abstract

Vascularization plays an important role in the microenvironment of the tumor. Therefore, it should be a key element to be considered in the development of in vitro cancer assays. In this study, we decellularized in vitro capillaries to remove genetic material and optimized the medium used to increase the robustness and versatility of applications. The growth pattern and drug responses of cancer cell lines and patient-derived primary cells were studied on decellularized capillaries. Interestingly, two distinct growth patterns were seen when cancer cells were grown on decellularized capillaries: “network” and “cluster”. Network formation correlated with the metastatic properties of the cells and cluster formation was observed in non-metastatic cells. Drug responses of patient-derived cells correlated better with clinical findings when cells were cultured on decellularized capillaries compared with those cultured on plastic. Decellularized capillaries provide a novel method for cancer cell culture applications. It bridges the gap between complex 3D culture methods and traditional 2D culture methods by providing the ease and robustness of 2D culture as well as an in vivo-like microenvironment and scaffolding for 3D cultures.

## 1. Introduction

The total cost of public cancer research worldwide is estimated at €7 billion per year, and in addition, the pharmaceutical industry spends €5.3–6.4 billion on cancer research [1]. Only about 10–14% of compounds in drug development have progressed successfully through clinical development [1,2]. By developing more reliable tools for research and drug development, these investments can more effectively produce new, safe treatment options for cancer patients.

Cell-based assays are an important pillar of the drug discovery process because they provide a simple, cost-effective, and rapid tool to avoid unnecessary animal testing of toxic or non-effective compounds. Since results are based on cells and their responses to compounds, these cell-based assays must be carefully developed and tested. The majority of cell-based assays still consist of monolayer cells cultured on flat and rigid substrates that do not take sufficient account of the natural environment of cells [3,4]. This has been noted, and in recent years more attention has been paid to the development of biomaterials that effectively support cell culture or cell transplantation with high cell viability or activity [5]. The difference in cell condition between in vivo and in vitro leads to low cell activity compared to in vivo, as cells in the body interact with other cells or extracellular matrix (ECM), leading to increased cellular activity in their differentiation, proliferation, metabolism, or cytokine secretion [5].

3D cell assays have been shown to have more in vivo-like cellular responses compared to 2D culture [6,7,8,9]. Also, in the field of cancer research, 3D models are being developed using various techniques, including chips, spheroids, and scaffold constructs [10,11,12]. New analysis methods are also being developed to allow easy analysis of the 3D methods, such as scanning tunneling microscopy techniques [13]. However, the disadvantages of 3D cell assays consisting of biomaterial and multiple cell types are that they often require long culture times and are difficult to modify and maintain. Therefore, they are not suitable for rapid screening of patient samples or for the propagation of rare patient-derived cells due to other contaminating cell types.

The interaction between blood vessels and cancer cells is vital for tumor growth and metastasis [14,15,16]. In addition, tumor angiogenesis may be the target of drug therapy, and the vascular functionality in tumors may determine the outcome of drug therapy through the entry of these drug molecules into the tumor site [15,17].

In vivo, the extracellular matrix (ECM) plays an important role in directing the cellular properties and responses [18]. The ECM of each tissue type has a unique composition and topology during development [19]. An in vivo-like ECM can be mimicked with biomaterials, such as Collagen or Matrigel. However, these commonly used alternatives are of animal origin, from a malignant source rather than normal tissue, or do not involve the complexity of the in vivo tissue ECM. In recent years, more and more studies have been conducted that focus on the correct composition of the ECM. Tissue-specific ECM has been studied in many research areas, including bone formation and regeneration [20] and tumor models [12], and the results have been promising. The correct composition of the ECM in cancer research is important because the ECM modulates the hallmarks of cancer progression and affects the proliferation of cancer cells [21].

We have previously developed human cell-based in vitro capillaries that can be used as a growth platform for primary cancer cell cultures [22]. These capillaries, which form a network of capillaries in culture, contain a lumen, an endothelial cell layer surrounded by contractile cells, and a basement membrane, including relevant attachment proteins found in in vivo vessels [23]. From now on, this whole construct is called “in vitro capillaries” and also contains supporting cells and ECM. Although the capillary component of the in vitro cancer cell culture is well justified due to the strong interaction between cancer cells and capillaries, living stromal cells (human adipose stromal cells, hASC) and endothelial cells (human umbilical vein endothelial cells, HUVEC) that form our in vitro capillaries, may cause some difficulties in the utilization of this living growth platform in some applications such as genetic studies and phase-contrast time-lapse imaging. In genetic studies, the heterogenous cell population that forms capillaries can cause problems in interpreting gene expression results. In phase-contrast imaging, target cells may not be distinguishable from capillary-forming stromal cells. To expand the range of applications, the aim of this study was to modify the in vitro capillary platform to better facilitate gene expression studies while preserving the capillary microenvironment produced by the cells themselves. We hypothesize that by utilizing decellularization, we could allow an in vivo- like tumor microenvironment for cancer cells and expand the application range of the culture method.

In this study, we decellularize the in vitro capillaries formed by hASC and HUVEC to develop a novel in vitro cancer cell culture method that can be used as a high-throughput, robust and easy-to-use platform for a wide variety of different cancer-related applications such as drug screening, drug development, and gene expression studies. The purpose of the study was, first, to determine whether cancer cells grow on decellularized in vitro capillaries (DC) and whether their morphology differs from that of plastic-growing cells. Second, we compared the responses and properties of cancer cells when grown on plastic or DC to determine if DC is a suitable growth platform for cancer cells.

## 2. Materials and Methods

### 2.1. Culturing of In Vitro Capillaries

hASC and HUVEC were isolated and quality controlled as described earlier [23,24]. The use of the tissue samples was approved by Ethics Committee of the Pirkanmaa Hospital District, Tampere, Finland, with permit numbers R15161 for hASC (dated 5 November 2015), and R15033 for HUVEC (dated 19 February 2015). Briefly, hASC were cultured in DMEM/F12 supplemented with 10% human serum (HS, Lonza Group Ltd., Basel, Switzerland) and 1% L-Glutamine (Gibco, Carlsbad, CA, USA). HUVEC were expanded in Endothelial Cell Growth Medium-2 BulletKit (EGM-2, Lonza). In the co-culture, hASC were at passage 2 and HUVEC at passage 4. Both cell types were free of mycoplasma tested with MycoAlert^®^ Mycoplasma Detection Kit (Lonza). hASC were characterized for markers CD73, CD90, and CD105 (BD biosciences, Franklin Lakes, NJ, USA) with flow cytometer FACSCAnto II (BD biosciences) before experimental use. In the co-culture, hASC were at passage 2 and HUVEC at passage 4.

The hASC and HUVEC coculture-based capillary network was established as published previously (angiogenesis model [23]) with a few modifications as follows. On day 0, hASC (20,000 cells/cm^2^) were seeded in EGM-2 (Lonza) followed by seeding of HUVEC (4100 cells/cm^2^) in EGM-2 (Lonza).

On day 1, the stimulation medium was added (Table 1). To increase the ECM production, macromolecular crowding (MMC) was utilized in the fabrication of in vitro capillaries by adding 50 µL of Ficoll-Paque PLUS (GE health care) per 1 mL of medium. The stimulation medium alone functioned as a control for the crowding. The stimulation media with or without crowder was replenished once during the 7-day culture. On day 7, the cultures were immunostained or decellularized as follows.

### 2.2. Decellularization

The decellularization protocol was modified from the protocol published by Ng et al. [25]. Before decellularization, the cultures were washed 3 times with PBS. Decellularization solution A (Table 1) was added; 120µL per well (a 96-well plate) and incubated for 5 min in 37 °C, followed by 2 washes with PBS and incubation in 60 µL decellularization solution B (Table 1) per well (a 96-well plate) for 30 min in 37 °C. After Solution B, 3 washes with PBS and plate storing at +4 °C.

The non-decellularized controls were fixed by incubating for 20 min in 70% ethanol at room temperature. At day 7, immunocytochemical and DAPI staining was performed.

### 2.3. Immunocytochemical Staining

For immunostaining, the cells were fixed on day 4 (all cell lines and all primary cells) or day 7 (samples 8–14 of the primary cells), depending on the growth of the cells, with 70% ethanol, permeabilized with 0.5% Triton-× 100 (MP Biochemicals), and treated with 10% bovine serum albumin (BSA, Roche) to block the non-specific binding sites prior to applying the primary and secondary antibodies. Primary antibody and secondary antibodies (Table 2) were applied in 1% BSA. A drop of Fluoroshield™ with DAPI histology mounting medium (Sigma) was added to stain the nuclei and mount the cultures.

### 2.4. Patient-Derived Tumor Cells

Tumor samples from 14 patients (Table 3), obtained for diagnosis or therapeutic indications, were used in this study: 11 liquid samples (ascites fluid and pleural effusion samples) and 3 solid tumor tissue samples. The use of the tissue samples and correlating data was approved by Ethics Committee of the Pirkanmaa Hospital District, Tampere, Finland, with permit number R16127 (dated 7 February 2017).

Isolation of cancer cells was performed as described earlier [22]. Briefly, liquid patient samples (pleural effusion or ascites fluid) were centrifuged at 200× *g* for 10 min, and cell pellets were washed, strained through 100 µm strainer, and resuspended to Liquid cancer sample medium (LCM, Table 1). These primary cancer cells were cultured a maximum of 6 days prior to the experiments.

Solid samples were isolated with Human tumor dissociation kit (Miltenyi Biotec, Bergisch Gladbach, Germany) according to manufacturer’s instructions. First, the tumor sample was manually dissected into 2–4 mm pieces. Pieces were placed into a gentleMACS C tube (Miltenyi Biotec) with enzyme mix provided in the kit. The tube was placed on a gentleMACS dissociator with heaters (Miltenyi Biotec). The samples were run with “37C_h_TDK_2” program, which is found in the gentleMACS dissociator. Cells were strained through a 70 µm MACS smartstrainer (Miltenyi Biotec). The suspension was centrifuged at 250× *g* for 7 min and resuspended into the general cancer cell medium (GCM, Table 1) also maximum of 6 days.

### 2.5. Culturing of Primary Cancer Cells and Cancer Cell Lines on the Decellularized In Vitro Capillaries

Cancer cells grown on the DC were analyzed for cell viability (also used as relative cell number, WST-1 analysis), drug sensitivity, and the growth pattern. Cells grown on plastic were utilized as control for the experiments. Each experiment with cell lines was repeated independently 3 times with at least 2 technical replicates (parallel wells on well plate) and with primary cells a minimum of 5 technical repeats (parallel wells on well plate) per analysis without independent repeats. The cell culture media used is shown in Table 1.

The testing of the system was performed with MCF7 cells, 9100 cells/cm^2^ on a 48-well plate in MCF7 culture medium (Table 1). Utilizing the results of the MCF7 experiences, further experiments were performed with PC3, LNCAP, ECC-1, U-87MG, A549, H460, PC3M, SH-SY5Y, KGN, 22RV1, ALVA-31 cell lines, and primary patient-derived cells. For further studies, 96-well plates were utilized, and seeding density was 15,150 cells/cm^2^, both on plastic and on the decellularized capillaries.

The day after cell seeding (day 1), the primary cell cultures were exposed to cancer drugs (Table 4). All samples were exposed to drugs for 3 days. Samples 8–14 were exposed for 3 and 6 days to also see the effect of longer exposure. Drugs were diluted in GCM (for solid tissue samples) or LCM (for liquid samples); see Table 1. The medium with drugs was replenished at day 4. Experiments included unexposed and vehicle control cultures. On day 4 (all cell lines and all primary cell samples) or on day 7 (samples 8–14 of the primary cells), the cultures were analyzed. The cell lines were cultured without drugs for up to 1 week to study their morphology and pattern formation on the decellularized capillary network. Cultures were not continued beyond 7 days.

### 2.6. WST-1 Analysis

The WST-1 assay was used for a spectrophotometric quantification of cell proliferation and viability. WST-1 (Roche) was incubated for 1.5 to 2 h. Absorbance was measured at 450 nm with a Varioskan flash multimode reader (Thermo Fischer Scientific, Waltham, MA, USA). When studying viability/proliferation (relative cell number), the WST-1 results were depicted as absorbance values.

In the drug exposure experiments, the absorbance of WST-1 was utilized to analyze the effectiveness of the studied drug. The drug was interpreted as “effective” if there was significantly lower viability in the drug-exposed cells than in the control. If no significant difference was found, the drug was classified as “not effective”.

### 2.7. Microscopic Analyses

Microscopic imaging was performed with Nikon Eclipse Ti-s inverted fluorescence microscope (Nikon, Tokyo, Japan) and Nikon digital sight DS-U2—camera (Nikon), and images were further processed with NIS Elements (Nikon), ZEN 2012 software (Carl Zeiss, Oberkochen, Germany). Automated imaging with 10 x objective was performed with Cell-IQ (CM Technologies Oy, Tampere, Finland). All image types were further processed using Adobe Photoshop CS3 software (Adobe Systems Incorporated, San Jose, CA, USA).

### 2.8. Statistical Analyses

The viability of the primary cancer cells on DC was analyzed with the Mann–Whitney test. The results of the cell lines grown on DC and plastic were subjected to the Kruskal–Wallis test with Dunn’s post-test. The drug response results were subjected to two-way ANOVA with Bonferroni post-test. Results are depicted as mean ± Standard deviation. Differences were considered significant when *p* < 0.05.

## 3. Results

### 3.1. Effect of Ficoll-Paque plus on In Vitro Capillaries

**Figure 1:**
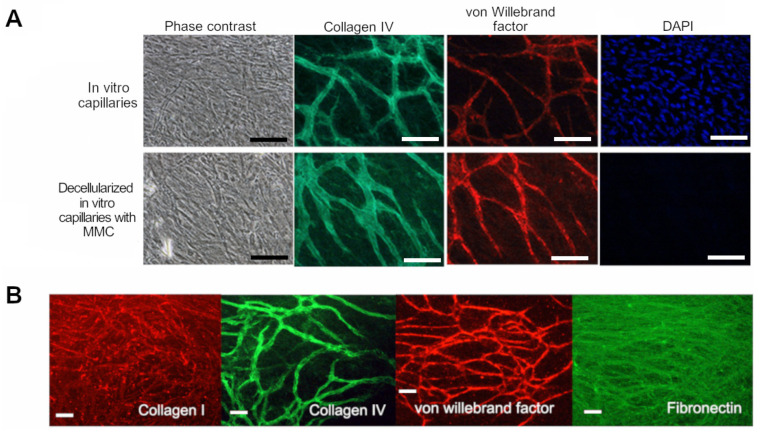
Immunofluorescence images of the proteins in Ficoll-Paque Plus crowded and decellularized in vitro capillaries. (**A**) Comparison of the in vitro capillaries without macromolecular crowder (MMC) or decellularization (top row) and Ficoll-Paque Plus crowded and decellularized in vitro capillaries (bottom row). The morphology of the culture imaged with phase contrast and expression of basement membrane marker Collagen IV-FITC (green), endothelial cell marker von Willebrand factor-A568 (red), and nucleus/DNA stain DAPI (blue). The capillaries with MMC have a thicker layer of Collagen IV, also these decellularized capillaries lack DNA seen as negative DAPI staining. (**B**) The staining of decellularized in vitro capillaries with MMC shows the presence of common ECM proteins fibronectin (green) and Collagen I (red), deposited by the hASC and HUVEC in the presence of macromolecular crowding. The endothelial cell marker von Willebrand factor-A568 (red) and basement membrane marker Collagen IV-FITC (green) are showing the localization of the decellularized in vitro capillaries. Decellularization has not removed these proteins. Immunostainings are performed with anti-von Willebrand factor visualized with A568 (red), anti-fibronectin (FITC, green), anti-Collagen I (A568, red), and Collagen IV (FITC, green). Scale bars 100 µm. Imaged with Nikon Eclipse Ti-s inverted fluorescence microscope.

To increase the deposition of ECM by the in vitro capillaries, we added Ficoll-Paque Plus (5%) as a macromolecular crowder to the medium. This provided good capillary network formation and was observed to increase deposition of Collagen IV and VWF, seen as thicker tubules in Figure 1A when compared to control. This observation was performed only by eye. Also, the addition of Ficoll-Paque Plus allowed a good accumulation of fibronectin and Collagen I in the culture (Figure 1B). The use of Ficoll-Paque Plus as a crowding agent was based on our previous study, in which we found that it did not disrupt the formation of in vitro capillaries when used with this concentration [26].

Due to the utilization of the macromolecular crowder, the growth factor concentration could be lowered to half of the previously published (now 5 ng/mL VEGF and 0.5 ng/mL FGF-β, respectively [23]), and still the amount of capillary network was the same.

### 3.2. Effects of Decellularization on Capillaries

Decellularized MMC capillaries were thicker than control capillaries (non-decellularized and not crowded) due to the increased ECM deposition induced by macromolecular crowding with Ficoll-Paque Plus. The decellularized capillary network showed negative staining for DAPI, i.e., did not contain DNA. The decellularized capillary network contained Collagen IV, von Willebrand factor, and ECM proteins fibronectin and Collagen I, as shown in Figure 1. This optimized, Ficoll-Paque Plus crowded and decellularized, in vitro capillary culture is hereafter referred to as decellularized in vitro capillaries (DC).

### 3.3. Decellularized Capillaries as Growth Platform for Cancer Cell Line Cells

**Figure 2:**
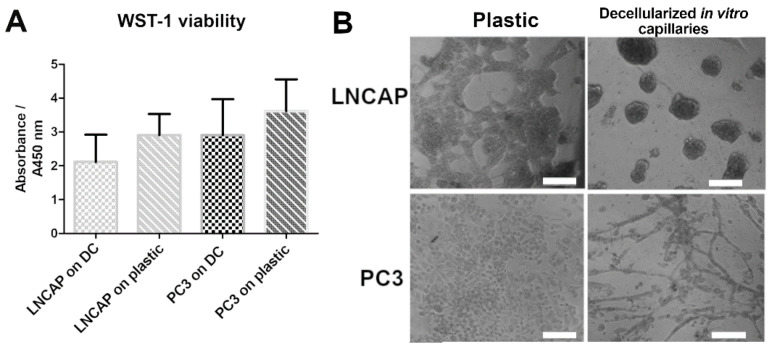
Cell lines grown on decellularized in vitro capillaries (DC). (**A**) WST-1 analysis of the proliferation of LNCAP and PC3 on DC and on plastic. No significant differences were seen between growth surfaces; however, the trend is that DC slows down the proliferation of the cancer cells. (**B**) Exemplary phase-contrast images of the cell lines LNCAP and PC3 grown on DC and on plastic. Both cell lines spread across the plastic surface. On DC, the LNCAP cells form round clusters, and PC3 cells align with the network of capillaries and they can be seen in phase contrast as a network. Scale bar 100 µm. Imaged with Nikon Eclipse Ti-s inverted fluorescence microscope.

Various cancer cell lines were grown on the DC and were studied for viability/proliferation, as well as growth patterns and localization on the DC. DC shows a trend of slowing the growth rate of LNCAP and PC3 compared to plastic (Figure 2). Results of other tested cell lines are collected in Table 5.

**Figure 3:**
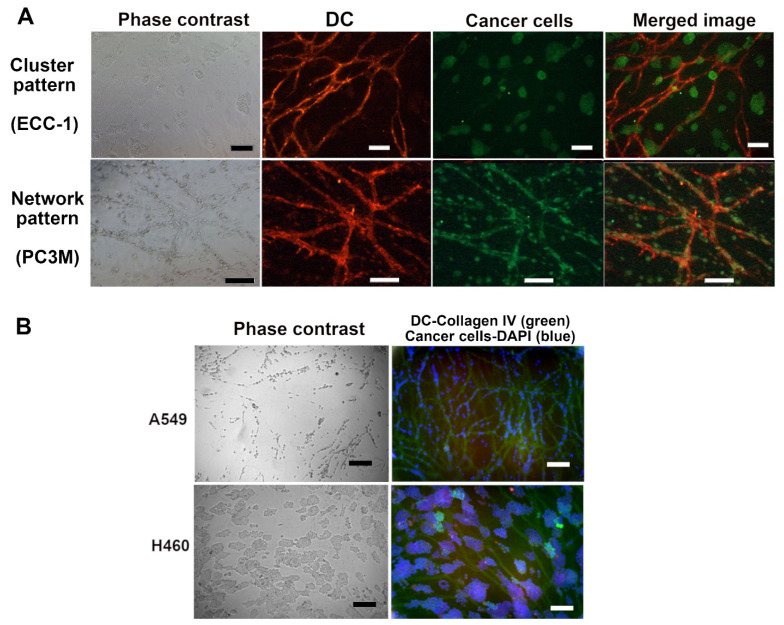
Growth pattern and immunostainings of cancer cell lines on decellularized capillaries. (**A**) Phase-contrast images show that SH-SY5Y forms loose clusters, ECC-1 forms clusters, and PC3M forms networks. Merged images show the co-localization of the network pattern (formed by cancer cells) with the DC. Clusters appear to localize more randomly by preferring proximity to capillary network. DC is stained with anti- von Willebrand factor (red) or with anti-Collagen IV (green). Cancer cells are stained with anti-α-Actin (green) or anti-ALDH1A1 (red). Imaged with Nikon Eclipse Ti-s inverted fluorescence microscope. (**B**) A549 co-localize with capillaries, and H460 cells form clusters. Left: the phase contrast image, right: the immunostained image of the culture; vascular basal membrane (Collagen IV, green) and Nuclei (DAPI, blue). Growth pattern of A549 (network) and H460 (cluster) are seen clearly in both images. Cancer cells are easily visualized with DAPI because capillaries no longer contain DNA. Scale bar 100 µm in all images.

The growth patterns of the cancer cells (monitored using phase-contrast microscopy) were compared to the cancer properties of these cells. We found that usually, the cell lines grew either (1) aligned with the capillary network forming a “network” pattern or (2) in roundish cell “clusters” when grown on DC. Figure 3 shows examples of the network and cluster patterns with two different staining options. Figure 3A shows prostate adenocarcinoma LNCAP forming clusters and prostate adenocarcinoma PC3 forming networks. Both cell lines formed a patternless monolayer on plastic. The location of the clusters varied, some were situated on the capillaries, and others grew on the capillary-free spaces in the culture vessel (Figure 3).

The decellularized platform allowed us to monitor the location of various cancer cells by utilizing DAPI staining for all different cell types (Figure 3B). This way, the search for cancer type-specific antibodies is avoided and experiments are more cost-effective. The goal of this study was to observe the patterns cancer cells take up and, as the patterns with cell lines were clearly one of the two categories, these were chosen as the pattern on which we focused. No quantification was found necessary for the growth patterns. However, a classification program was being developed to automatically recognize whether the specific cancer cell sample takes up the network or cluster formation [53]. This was performed from phase-contrast images. The visualization of the growth patterns is included in Supplemental Figure S1. In this study, the classification was performed by eye via microscopic inspection.

### 3.4. Drug Responses of Cancer Cells Grown on Decellularized In Vitro Capillary Network

Patient-derived samples were tested for survival and growth patterns on DC in similar fashion as cell line cells. The patient-derived cell population in this study was heterogenic and contained cells isolated from liquid samples (pleural effusion or ascites fluid) and solid tumor samples. The goal of this study was to see whether the DC is a suitable growth platform for cancer cells regardless of their specific type or origin. In addition, the heterogenic sample pool was needed to investigate which cell types and properties are contributing to the “network” and “cluster” pattern formation. By having multiple cancer and sample types, we could better see if some cells do not grow on DC and if the drug sensitivity results obtained on DC depend on specific cancer properties. Cells from all 14 samples were recovered successfully. The patient-derived cells were grown on the DC and plastic for a maximum of 7 days.

**Figure 4:**
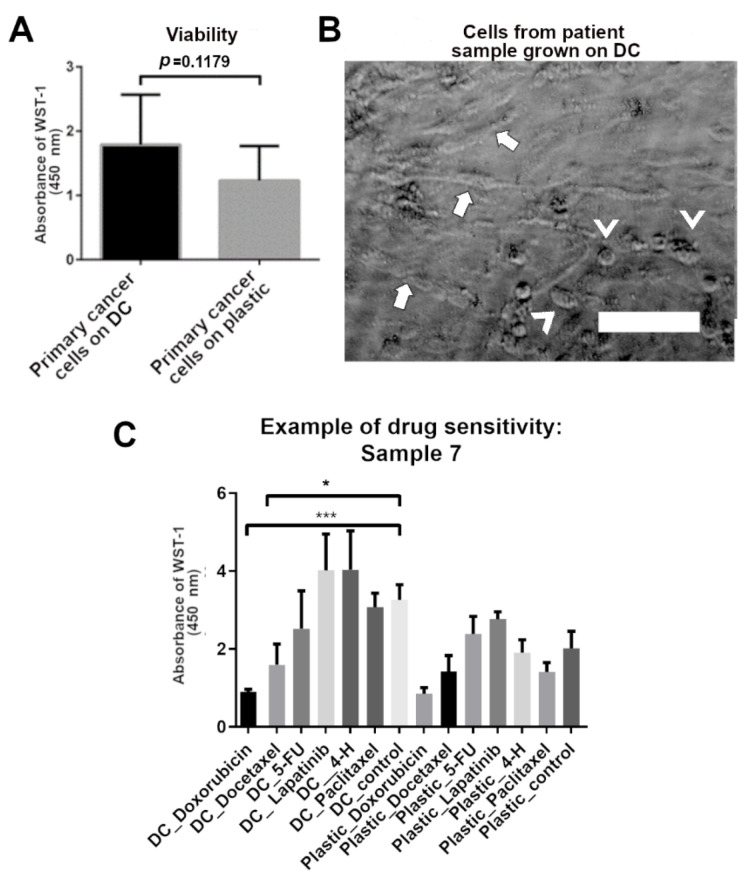
Primary cancer cells on decellularized in vitro capillaries (DC). (**A**) WST-1 analysis results of the proliferation of all patient-derived cells on DC and on plastic. No significant differences were seen between growth surfaces; however, the trend is that patient-derived cells grown on DC have higher viability. Mean ± SD is shown. (**B**) A phase-contrast image of one patient sample, sample 7, showing “network” pattern in the culture (marked with arrows). Clusters formed by patient-derived cells are marked with arrowheads. Scale bar 100 µm. Imaged with Cell-IQ. (**C**) Exemplary image of the drug sensitivity of sample 7 showing the results with only the highest concentration of drugs tested. When cultured on plastic, the cells did not show sensitivity towards any of the drugs utilized in the study. When cultured on DC, the cells showed sensitivity to doxorubicin (6 µM) and docetaxel (3 µM). *** *p* ≤ 0.001, * *p* ≤ 0.05. 5-FU = 5-fluorouracil, 4-H = 4-hydroperoxycyclophosamide (in vitro active metabolite of cyclophosamide).

The results show that there was no significant difference in the viability of primary cells grown on the DC compared to those grown on plastic (Figure 4). However, the viability of primary cells seems to be higher on DC on average. The growth patterns seen in cell lines also existed in cancer cell lines but were not as easily recognizable as they were in cell lines. Figure 4B shows the “network” pattern and “cluster” pattern of primary cell culture. This sample was classified as having both cluster and network (Table 6). When classifying the growth patterns of primary cell samples, if no network pattern was seen, the samples were categorized as “cluster” or “no pattern”. The classifications were performed by eye via phase-contrast microscopy.

To test the drug sensitivity of the patient samples, patient-derived cells were challenged with doxorubicin, docetaxel, 5-fluorouracil, lapatinib, 4-hydroperoxycyclophosamide (in vitro active metabolite of cyclophosamide), and paclitaxel. The viability was measured after three-day or six-day drug treatment. Culturing on DC was associated with increased sensitivity to drugs after three days of drug treatment compared to cells grown on plastic. Various drug responses were seen in patient samples (Table 6). Figure 4C shows exemplary drug sensitivity results of one patient sample (sample 7) with the highest tested concentrations. Doxorubicin and docetaxel were found effective most often. No correlation between diagnosis and doxorubicin and docetaxel sensitivity was found. Some patient samples had the same results on plastic and on DC. These samples represented various cancer types and disease grade/stages. Some samples were more sensitive to drugs on DC and some on plastic. More sensitive samples were lung and colon adenocarcinomas (progressive and stable), mammary carcinoma, ovarian cancer, and thyroid cancer. Pleural effusion samples seemed to contain more sensitive cells on DC than on plastic. The results of the drug sensitivity experiments show a good correlation with the patient response data available (Table 6).

In contrast to cell lines, the growth patterns of patient-derived cells seem to correlate more with the drug sensitivity than disease data (Table 6). With patient samples, those forming only clusters showed higher sensitivity to drugs on DC. Network patterns occurred more likely with cells that responded to drugs in a similar manner both on DC and plastic. In this study, the interpretation of the drug sensitivity correlation is incomplete as the laboratory did not have all the cancer drugs in use that are used by the clinicians. Hence, patients had treatments that could not be tested on the cells in the laboratory. Hence, the correlation is based on the information and results available (Table 6).

## 4. Discussion

In this manuscript, we present a novel alternative to traditional 2D cultures (cells on plastic) for growing primary cancer cells and cancer cell lines. In this study, we decellularize the in vitro capillaries formed by hASC and HUVEC to develop a novel in vitro cancer cell culture method. This can be utilized as a high-throughput, robust and easy-to-use platform for a wide variety of cancer-related applications, such as drug screening, drug development, and gene expression studies.

To obtain the maximum amount of ECM, we used macromolecular crowder Ficoll-Paque Plus during the cultivation of the vascular network. DC was studied by immunocytochemistry to ensure that relevant vascular markers were still present after the decellularization process. Although characterization of vascular markers was minimal, including only Collagen IV and VWF, the functional results (the fact that cancer cells find the decellularized capillary structures) confirm that the required proteins are still present after the decellularization. We saw that the morphology of the vascular network remained intact and that the DC lacked DAPI staining, i.e., DNA removal was successful. The lack of DNA in DC allows for easier genetic studies on this platform, allowing easy analysis of cells grown on DC.

ECM composition plays an important role in cancer research. ECM appears to affect tumor proliferation and metastasis [21,54]. Majumder et al. showed that tumor explants grown in uncoated wells lost tumor architecture and showed reduced viability, proliferation, and activation of oncogenic pathways [55]. Explants cultured on cancer type-specific tumor-stromal matrix proteins retained tumor morphology, viability, proliferation, and phospho-ERK1/2 status [55]. The ECM in DC is produced by the hASC and HUVEC during the vascular network formation. The deposition of Collagen I, Fibronectin, and Collagen IV found in DC was enhanced by the addition of the macromolecular crowder, Ficoll-Paque Plus, and the use of albumin (BSA) as the second macromolecule in the medium. ECM components allow attachment sites for DC-grown cells, and no additional biomaterial coating or gel is required for the cultivation of the cells. Tumors have blood vessels, and the ECM provided by the blood vessels is important in providing the right ques for the cancer cells. The results confirm that DC is a suitable culture platform for cancer cells because it provides a more in vivo-like microenvironment for cancer cells. Because DCs do not have living cells, DC experiments can be designed according to the requirements of the cancer cells, and parameters such as culture time can be adjusted according to the needs of the cells in question. The scalability of the vascular network [22] also allows the use of DC in high-throughput studies.

Decellularization has provided the cells with a natural support structure that has been used in clinical applications [56,57,58], including soft tissue regeneration [59]. In the in vitro environment, the decellularization approach has already been tested with liver tumor constructs. The decellularized liver matrix was used in a tumor-on-a-chip model developed for toxicity testing, and the results were promising for achieving an in vivo-like tumor microenvironment in an in vitro culture environment [12]. Similarly, in the present study, decellularized in vitro capillaries were used as a scaffold for cancer cells. By decellularizing the capillary network, the genetic material, as well as the medium requirements of the system, were removed. Furthermore, the immune system modulating properties of hASC [60] were removed, allowing the use of DC in applications related to immune-oncology.

The effect of DC on the cellular properties and responses of cancer cells was tested by growing cancer cell lines and primary cancer cells on DC. One of the main findings of this study was that the cell lines grown on DC were found to organize into two distinct growth patterns, i.e., “a cluster” and “a network”, which were visible by phase-contrast microscopy. In the network pattern, the cells aligned with the capillary network. Those cells that were attracted to decellularized capillary structures are cells that would metastasize in vivo, according to literature (Table 5). The cells that formed the “cluster” pattern were less metastatic and less tumorigenic, according to the literature. In cases where the cell line formed both a “network” and a “cluster” pattern, such as prostate adenocarcinoma ALVA-31 and prostate adenocarcinoma PC3M, cell lines have been reported to be metastatic in the literature. Ovarian granulosa cell carcinoma KGN and Neuroblastoma SH-SY5Y were classified in the literature as invasive/metastatic cell lines but did not form a network pattern when grown on DC. Non-small cell lung cancer A549, ALVA-31, PC3M, glioblastoma U87-MG, and PC3 were classified in the literature as metastatic cell lines and adapted the network growth pattern on DC (Figure 3, Supplemental Figure S1 and Table 5). Prostatic carcinoma 22RV1, endometrial adenocarcinoma ECC-1, LNCAP, breast ductal carcinoma MCF7, and large cell cancer of the lung H460 were classified as non-metastatic cell lines in the literature and adapted the “cluster” growth pattern on DC. The type or origin of the cancer did not appear to affect the growth pattern. The cell line forming both the “network” and “cluster” patterns was reported to be metastatic in the literature. Based on these findings, it seems that when the “network” pattern is seen, the cell has metastatic properties. The growth rate of cell lines on DC also appeared to be more limited, mimicking the growth of these cancer cells in vivo better than the rapid proliferation of these cell line cells on plastic, spreading across the culture vessel in an evenly distributed monolayer.

For the patient-derived cancer cells, we saw the same “network” and “cluster” growth patterns on DC, but this was not as clear as with the cell lines. Thus, with further optimization, this platform could help predict patient-specific metastatic potency of cancer. We also found that primary cells showed a trend of higher viability on DC than on plastic. Successful culturing of various primary cancer cells suggests that even rare and more difficult patient-derived cancer cells could be cultured in vitro on DC. Such cell types can be circulating tumor cells (CTC), prostate cells, or any biopsy containing only a small number of primary cancer cells [61,62,63]. Only a few successes have been achieved in establishing immortalized CTC cancer cell lines [64,65,66] or invasive human pancreatic cancer cell lines [67]. Very common and lethal prostate cancer has also shown to be difficult to maintain unmodified ex vivo [68,69].

The attraction of the metastatic cell lines to the capillary network can be linked to the importance of vasculature in the growth and spreading of cancer. Tumor cells have been shown to co-opt, migrate along, and proliferate on the surface of host vessels [70]. Vascular co-option is common in carcinomas originating from single epithelia (e.g., many types of breast cancer), in tumors of mesenchymal origin (sarcomas) [71], and in glioma cell lines that have been shown to utilize vascular/vessel co-option as blood supply method [72]. This also correlates with the “network” growth pattern seen on DC for the glioma cell line U87-MG, as they localized on the decellularized capillary structures. Cells lacking a metastatic tendency seemed to be less attracted to vasculature because clusters localized more randomly in the cultures. Based on our findings, DC could well indicate tumor types using vascular co-option. This, in turn, could help in choosing the right treatment for the patient between anti-angiogenenic treatment and other treatment options.

Interestingly, our results on the growth patterns of cell lines are in good agreement with the results of a previously published 3D multicellular spheroid study [73]. Härmä et al. formed spheroids out of PC3, PC3M, ALVA-31, LNCAP, and 22Rv1. They classified PC3, PC3M, and ALVA-31 as stellate-forming cell lines in which stellates grow out of spheroids. LNCAP and 22Rv1 represented mass cell lines that formed round spheroids without stellates [73]. PC3, PC3M grew in the “network” pattern, ALVA-31 cultures contained both the network and the cluster and LNCAP and 22Rv1 the “cluster” pattern on DC. This result is promising for our goal of developing a cancer model that allows the formation of different growth patterns similar to 3D spheroids but still includes the benefits of 2D culture, such as the robustness and ease of imaging. Glioma cell lines have been shown to utilize vascular/vessel co-option as a blood supply method [72]. This also correlates with the “network” growth pattern seen on DC for the glioma cell line U87-MG. These cells migrate to decellularized vascular structures, indicating that the critical proteins are still in place in DC structures.

The pattern formation in primary cells was not classified as easily as in cell lines. In contrast to cell lines, the growth patterns of patient-derived cells appear to correlate more with drug sensitivity than with disease data (Table 4). Samples that form “clusters” on DC showed greater sensitivity to drugs, whereas a “network” pattern was more likely to be present in cells that responded similarly to drugs on both DC and plastic. No correlation was observed between the growth pattern of patient-derived cells and tumor location, cancer grade, or stage of the tumor.

Automated pattern recognition software was developed for the automatic classification of growth patterns seen on DC [53]. The possibility to directly analyze phase-contrast images is a great advantage because it is cost-effective and less laborious than immunostaining. It also allows live monitoring of cells without compromising cell-ECM interactions with exogenous reagents such as live probes and stains.

Although more extensive testing of this novel decellularized vascular network is needed, the results presented here indicate that this is the right option to bring 2D cultures closer to the in vivo growth environment. Extensive research on drug responses on this platform in patient cells with more extensive ethical approvals would be crucial to validate the correct responses of cancer cells. For more efficient assessment of the sensitivity of the patient samples to different drugs, it would be important that the same set of drugs is available for both clinicians and research laboratory. The results presented in this study show that DC is a promising growth platform for cancer cells.

## 5. Conclusions

Decellularized in vitro capillaries (DC) provide a novel tool for in vitro cancer cell research by providing an in vivo-like vascular surface for cancer cell growth. DC can be considered as a bridge between complex 3D culture methods and traditional 2D culture methods by providing the ease and robustness of 2D culture as well as the in vivo-like tumor microenvironment of more complex 3D cultures. Decellularization of the in vitro capillaries allows easy use and storage of this novel culture platform. DC contains (1) a natural, human cell-produced ECM and (2) a capillary network with vascular surface proteins that provides a 3D growth surface for different cell types. As such, it can be used in a variety of cell culture applications.

## Figures and Tables

**Table 1:** Composition of media and solutions used in the study and their manufacturers.

Medium	Composition	Manufacturer
Stimulation medium	DMEM/F12 2.56 mM L-glutamine 0,1 nM 3,3’,5-Triiodo-L-thyronine sodium salt (T3) ITS^TM^ Premix: 6.65 µg/mL insulin 6.65 µg/mL Transferrin 6.65 ng/mL seleniuous acid 1% Bovine serum albumin (BSA) 2.8 mM Sodium puryvate 100 µg/mL Ascorbic acid (AA) 0.25 µg/mL Heparin (HE) 1 µg/mL Hydrocortisone/cortisol (HY) 5 ng/mL Vascular endothelial growth factor (VEGF) 0.5 ng/mL fibroblast growth factor (FGF-β)	Gibco, Carlsbad, CA, USA Gibco Sigma (Saint Louis, MO, USA) BD (Franklin Lakes, NJ, USA) PAA (Pasching, Austria) Gibco Sigma Sigma Sigma R&D Systems (Minneapolis, MN, USA) R&DSystems
Decellularization A solution	0.5% Triton X-100 in 0.02 M NH_4_OH with 0.5 × Complete Protease inhibitor without EDTA	MP Biochemicals, (Solon, OH, USA) Honeywell Fluka (Regen, Germany) Roche (Basel, Switzerland)
Decellularization B solution	30 U/mL DNase and 0.5 × Complete Protease inhibitor without EDTA in 1 × DNAse Buffer	New England Biolabs (Ipswich, MA, USA) Roche New England Biolabs
General cancer cell medium (GCM)	DMEM/F12 2 mM L-glutamine 5% Human serum	Gibco Gibco Lonza
Liquid cancer sample medium (LCM)	DMEM/F12 2 mM L-glutamine 10% Supernatant from the isolation of the cells	Gibco Gibco
MCF7 medium	DMEM/F12 2 mM L-glutamine 10% Fetal bovine serum (FBS) 10 ng/mL insulin	Gibco Gibco Gibco Sigma
SH-SY5Y, KGN	DMEM/F12 2 mM L-glutamine 10% FBS 100 U/mL penicillin, 100 µg/mL streptomycin	Gibco Gibco Gibco Gibco
U87-MG	EMEM 2 mM L-glutamine 10% FBS 1% NEAA 1 mM Sodium puryvate	ATCC (Manassas, VA, USA) Gibco Gibco Gibco Gibco
PC3, LNCAP, and PC3M, 22RV1, ALVA-31, ECC1 medium	RPMI1640 (containing 1 mM L-glutamine) 10% FBS 100 U/mL penicillin, 100 µg/mL streptomycin	Gibco Gibco Gibco
A549 medium	DMEM 2 mM L-glutamine 10% Fetal calf serum (FCS) 100 U/mL penicillin, 100 µg/mL streptomycin	Gibco Gibco Gibco Gibco
H460 medium	RPMI1640 2 mM L-glutamine 10% FCS 100 U/mL penicillin, 100 µg/mL streptomycin	Gibco Gibco Gibco Gibco

**Table 2:** Antibodies utilized in the study, their targets, and manufacturers.

Antibody, Product Number	Target	Manufacturer
Anti-human von Willebrand factor IgG (anti-VWF), F3520	Endothelial cells	Sigma
Anti-collagen IV (anti-COLIV), clone COL-94, C1926	basement membrane	Sigma
anti-ALDH1A1, ab52492	cancer cells	Abcam
anti-α-actin, A7811	cancer cells	Sigma
anti-fibronectin, ab194395	ECM	Abcam
anti-collagen I, SAB4500362	ECM	Sigma
anti-rabbit IgG A568, A11011	secondary antibody	Invitrogen
anti-mouse IgG fluorescein isothiocyanate (FITC), F4143	secondary antibody	Sigma

**Table 3:** Primary tumor samples and available information. N/A = not available or unclear.

**Sample**	Cancer Type	Sex	Race	Sample	Progressive Disease	Grade/Stage
Sample 1	Hepatocellular carcinoma	Male	Caucasian	Ascites fluid	Yes	Metastasized
Sample 2	Lung adenocarcinoma	Male	Caucasian	Pleural effusion	N/A	Metastasized
Sample 3	Lung adenocarcinoma	Male	Caucasian	Pleural effusion	Yes	Metastasized
Sample 4	Carcinoma ventriculi	Male	Caucasian	Pleural effusion	N/A	Metastasized
Sample 5	Mammary carcinoma	Female	Caucasian	Pleural effusion	N/A	Metastasized
Sample 6	Gastrointestinal adenocarsinoma	Male	Caucasian	Ascites fluid	N/A	Metastasized
Sample 7	Ovarian cancer	Female	Caucasian	Solid tumor	N/A	High grade, localized
Sample 8	Originating from colon, adenocarcinoma	Male	Caucasian	Ascites fluid	Yes	Metastasized
Sample 9	High-grade serous epithelial ovarian cancer	Female	Caucasian	Ascites fluid	Yes	High grade
Sample 10	Thyroid cancer	Male	Caucasian	Pleural effusion	Yes	Metastasized
Sample 11	Ovarian cancer	Female	Caucasian	Solid tumor	N/A	Localized
Sample 12	Breast cancer	Female	Caucasian	Pleural effusion	Yes	Metastasized
Sample 13	Sigmoidal adenocarcinoma	Female	Caucasian	Ascites fluid	Yes	Metastasized
Sample 14	Ovarian cancer	Female	Caucasian	Solid tumor	N/A	Localized

**Table 4:** Cancer drugs utilized in the study and their concentrations.

Cancer Drug	Concentrations Used in The Study
Doxorubicin	6 µM, 3 µM or 0.3 µM
Docetaxel	3 µM, 1 µM or 0.1 µM;
5-fluorouracil	6 µM, 3 µM or 1 µM
Lapatinib	6 µM, 3 µM or 1 µM
4-hydroperoxycyclophosamide (active metabolite of cyclophosphamide)	100 µM, 10 µM or 1 µM
Paclitaxel	6 µM, 3 µM or 1 µM

**Table 5:** Proliferation and pattern formation of the tested cell lines on decellularized in vitro capillaries (DC). Metastatic cell lines are presented on grey background and non-metastatic cell lines on white background. *n* = 3. N/A = data not available.

	Cell Line	Origin/Description	Tumorigenicity of the Cells	3D Culture Pattern from Literature	Reference	Growth Pattern on DC	Proliferation on DC vs. Plastic
Metastatic/invasive cell lines	A549	Non-small cell lung cancer	Tumorigenic	N/A	[27]	Network	N/A
	ALVA-31	Prostate adenocarcinoma, metastasis from bone	Tumorigenic	Invasive in 3D culture	[28,29,30,31]	Both clusters and network	Lower on DC, non-significant
	KGN	Invasive ovarian granulosa cell carcinoma, stage III	Tumorigenic, slow tumor growth	N/A	[32,33]	Not forming specific pattern	No difference
	PC3M	Prostate carcinoma, derived from PC3	High	Invasive in 3D	[29,34]	Network, some clusters	Faster on DC
	SH-SY5Y	Neuroblastoma, metastatic bone tumor	Tumors in nude mice in 3–4 weeks	N/A	[35,36]	Loose cluster	No difference
	U87-MG	Glioblastoma	High tumorigenic	N/A	[37]	Network	No difference
	PC3	Prostate adenocarcinoma, bone metastasis grade IV	High tumorigenic	Invasive in 3D	[29,38,39]	Network	Lower on DC, non-significant
Non- metastatic cell lines	22RV1	Prostatic carcinoma xenograft line, derived from CWR22R	Tumorigenic	N/A	[40,41]	Unevenly shaped large clusters	Lower on DC, non-significant
	ECC-1	Endometrial adenocarsinoma, grade 2	Well-differentiated, low proliferation	N/A	[42,43,44,45]	Cluster	Lower on DC, non-significant
	LNCAP	Human prostate adenocarcinoma, lymph node metastasis	Low tumorigenic	Non-invasive in 3D	[29,46,47]	Cluster	Lower on DC, non-significant
	MCF7	Breast ductal carcinoma, pleural effusion	Low tumorigenicity without estrogen	N/A	[48,49,50]	Cluster (no estrogen supplementation used)	No difference
	H460	Large cell cancer of the lung	Low tumorigenic potential	N/A	[51,52]	Cluster	N/A

**Table 6:** The results from the patient-derived cancer cells. The growth patterns formed by the patient-derived cancer cells and responses of patient-derived cancer cells to selected drugs on plastic and on DC (after three-day or six-day treatment). All samples were analyzed after 3-day drug exposure and samples 8–14 also after 6-day drug exposure. Clinical data on the metastatic state and drug sensitivity is also listed. The correct correlation between results on DC and clinical responses is on green background. *** *p* ≤ 0.001, ** *p* ≤ 0.01, * *p* ≤ 0.05.

Sample/Type	Growth on DC	Metastatic Cancer?	Drug Sensitivity on DC	Drug Sensitivity on Plastic	Clinical Drug Sensitivity	Responses on DC Correlate with Clinical Observations?
1/A	No clear pattern	Metastasized, PD	None	None	No effective drugs known	Yes: no effective drugs known
2/PE	No clear pattern	Metastasized	Doxorubicin ***, Docetaxel *	None	EGFR negative (no response for lapatinib), doxorubicin and docetaxel, commonly used for this cancer type	Yes: Doxorubicin and docetaxel commonly used, lapatinib not effective
11/S	No clear pattern	Localized	D3: Doxorubicin ***, D6: doxorubicin ***, docetaxel ***, paclitaxel ***	D3: Doxorubicin ***, D6: Doxorubicin ***, docetaxel **, paclitaxel ***	No treatment received	Unknown
3/PE	Cluster	Metastasized, PD	Doxorubicin **	None	EGFR neg (not responsive to lapatinib) treated with cisplatin	Yes: lapatinib not effective
8/A	Cluster	Metastasized, PD	D3: Doxorubicin *** D6: Doxorubicin ***, docetaxel ***, lapatinib ***, paclitaxel ***, 5-FU ***	D3: None D6: Doxorubicin ***, docetaxel ***, paclitaxel **, 5-FU ***	Not responsive to oxaliplatin or anti-angiogenic regorafenib	Unkown
9/A	Cluster	High grade, PD	D3 and D6: Doxorubicin ***, Docetaxel ***, paclitaxel ***	D3 and D6: Doxorubicin ***, Docetaxel **, paclitaxel ***	Not responsive to paclitaxel	No
10/PE	Cluster	Metastasized, PD	D3 and D6: Doxorubicin***	D3 and D6: none	Not responsive to anti-angiogenic sorafenib	Yes: anti-angiogenic lapatinib not effective
12/PE	Cluster	Metastasized, PD	D3: Doxorubicin ***, docetaxel ***, lapatinib *** D6: doxorubicin ***, paclitaxel ***	D3: None, D6: Doxorubicin ***, paclitaxel *, docetaxel **	ER+, PR+, HER2−, Not responsive to Cabecitabine (5-FU)	Yes: Capecitabine not effective
4/P	Cluster and network	Metastasized	Doxorubicin **	Doxorubicin **	No response for 5-FU or capecitabine. Doxorubicin could be effective for this cancer	Unknown: Doxorubicin could be effective by clinicians estimate
13/A	Cluster and network	Metastasized, PD	None	None	Not responsive to Cabecitabine	Yes: Capecitabine not effective
7/S	Cluster and network	High grade, localized	Doxorubicin ***, Docetaxel *	None	Naive sample, responsive to paclitaxel, docetaxel	Yes: docetaxel effective on DC
14/S	Cluster and network	Localized	D3: Doxorubicin *** D6: Doxorubicin ***, Docetaxel **, paclitaxel ***	D3: Doxorubicin D6: Doxorubicin ***, paclitaxel ***	Paclitaxel should be effective	Yes: Paclitaxel effective
5/PE	Network	Metastasized	Doxorubicin ***, Docetaxel **	Doxorubicin ***	ER+ PR+, HER2−, Not responsive to Docetaxel	No
6/A	Network	Metastasized	Doxorubicin ***, Docetaxel ***	Doxorubicin ***, Docetaxel ***, 5-FU ***, Capecitabine *, Lapatinib **	Resistant to capesitabine (5-FU pro-drug)	Yes: Capesitabine not effective on DC

S = solid, PE = pleural effusion, A = ascites, PD = progressive disease, 5-FU= 5 fluorouracil, D3 = three-day treatment, D6 = six-day treatment.

## Data Availability

The data are available from the authors upon request with some privacy and ethical restrictions. Tissue sample donors of this study did not agree for their identifying medical data to be shared publicly.

## Supplementary Materials

The following supporting information can be downloaded at: https://www.mdpi.com/article/10.3390/biomedicines10020271/s1, Figure S1: Phase contrast image of all the studied cell lines grown on decellularized in vitro capillaries (DC).

## Abbreviations

BSA	Bovine Serum Albumin
CTC	Circulating tumor cells
DC	Decellularized in vitro capillaries
ECM	Extracellular matrix
EGM-2	Endothelial Cell Growth Medium-2 BulletKit
FBS	Fecal bovine serum
FCS	Fecal calf serum
FGF-β	Basic fibroblast growth factor
GCM	General cancer medium
hASC	Human adipose stromal cell
HUVEC	Human umbilical vein endothelial cell
LCM	Liquid cancer sample medium
MMC	Macromolecular crowder
VEGF	Vascular endothelial growth factor
VWF	Von Willebrand factor
