# Supplementary material for: Decellularized In Vitro Capillaries for Studies of Metastatic Tendency and Selection of Treatment
Source: Biomedicines. 2022 Jan 26;10(2):271. doi: 10.3390/biomedicines10020271 (PMC8869401; doi:10.3390/biomedicines10020271)
Supplement: Supplementary file 1 [file biomedicines-10-00271-s001.zip › biomedicines-1537261-supplementary.pdf]

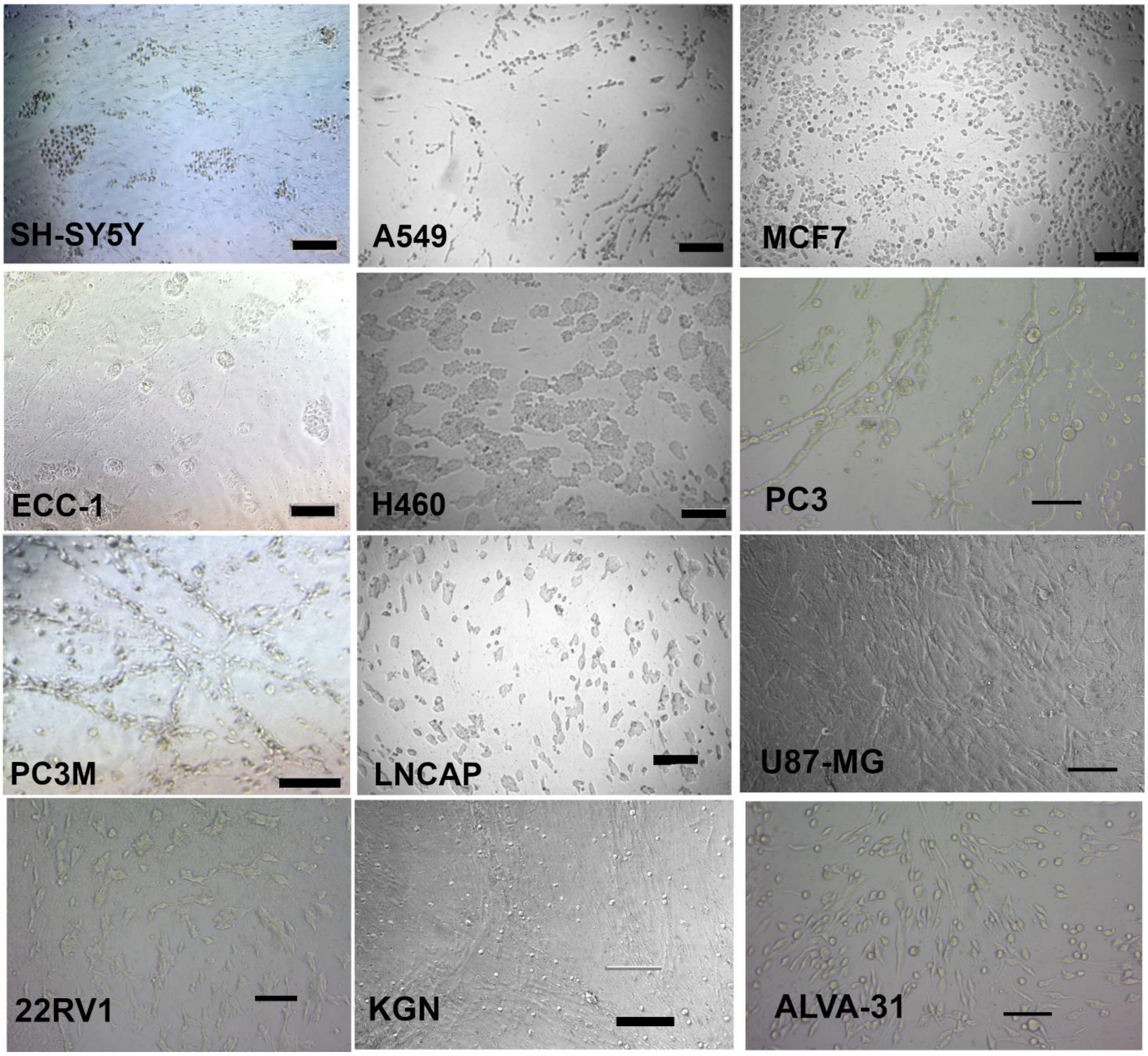

Supplemental Figure S1. Phase-contrast image of all the studied cell lines grown on decellularized *in vitro* capillaries (DC). The growth patterns of these cell lines on DC, mainly either "network" and "cluster" can be seen from the images. Scale bar 100 μm in all images.
